# Environmental and energy analysis of chromium recovery from residual tanned leather using alkaline thermal hydrolysis

**DOI:** 10.1038/s41598-024-84726-0

**Published:** 2025-04-02

**Authors:** Shima Shafiei Ahmadi, Mohammadali Maysami, Reza Abdi, Mahmoud Zarei, Stefan Dröge

**Affiliations:** 1 Department of Biosystems Engineering, Faculty of Agriculture, University of Tabriz, Tabriz, Iran; 2 Department of Applied Chemistry, Faculty of Chemistry, University of Tabriz, Tabriz, Iran; 3 Prüf- und Forschungsinstitut Pirmasens e.V., Department of Biotechnology, Marie-Curie-Str. 19, D-66953 Pirmasens, Germany

**Keywords:** Leather waste, Chromium recovery, Energy recovery, Life cycle assessment, Cumulative energy demand, Climate-change ecology, Environmental biotechnology

## Abstract

The leather industry efficiently uses livestock byproducts but struggles with pollution, especially from chromium in waste. Innovations in chromium recovery can prevent contamination and offer economic benefits, aligning with circular economy principles. However, environmental assessments like life cycle assessment (LCA) are crucial for sustainability. This study evaluates the environmental and energy implications of chromium recovery from leather waste using LCA. Findings indicate that recovering 1 kg of chromium through thermal hydrolysis with an alkaline method results in $ 8.42E-02 resource damage, 4.28E-06 DALY to human health, and 1.60E-08 species year ecosystem damage, according to the ReCiPe method. Sodium hydroxide significantly contributes to environmental damage, highlighting the need for sustainable strategies. With a weighted impact of 201.04 mPt/kg, human health accounts for 62% of the burden, and resource depletion 34%. Recovered chromium reduces environmental damage by 95.65% overall compared to raw production, demonstrating substantial sustainability benefits. The energy assessment shows sodium hydroxide dominates consumption, using 98% of total demand, with 98% from non-renewable sources. Despite energy challenges, chromium recovery reduces environmental impact compared to crude production, promoting ecological resilience.

## Introduction

The leather industry, a longstanding enterprise, transforms waste products from the food industry into a diverse array of consumer goods like shoes, garments, and bags, rendering them desirable, useful, and sustainable^1^. Over recent decades, global production has reached approximately 1.7 billion square meters of leather products annually, valued at around 40 billion dollars. Each year, approximately 6.6 million tons of hides from domestic cattle and 0.8 million tons of skins from sheep are converted into valuable leather materials^2^. This industry holds significant economic importance worldwide, with a global trade value estimated at approximately US$100 billion annually. Notably, it plays a crucial role in the economies of several developing countries, particularly those with the largest cattle herds such as Brazil, China, and India^3^. By utilizing raw materials effectively, the leather industry generates substantial economic benefits while addressing potential pollution issues associated with waste from the meat industry^4^.

Despite its significance, the leather industry is often categorized as highly polluting, with concerns regarding its adverse environmental impact^5^. Currently, the waste generated by this industry places it in the “red” category, indicating severe environmental implications^6^. Leather manufacturing involves a chemical process utilizing a substantial amount of water and various inorganic and organic chemicals, leading to the discharge of solid and liquid wastes^7^. Solid waste produced includes trimmings, fleshing, shavings, buffing dusts, and keratin wastes, with significant quantities generated per unit of hides or skins processed^8^. Unfortunately, a large portion of leather waste is sent to landfills, resulting in no material or energy recovery and causing significant environmental harm. Moreover, wastewater sludges released by leather industries contain toxic heavy metals such as chromium, copper, cadmium, and zinc, further exacerbating pollution concerns^2^. The effluent from tannery operations is particularly problematic, contributing to severe pollution in the surrounding environment^9^. More specifically, chromium tanned leather is celebrated for its versatility, hydrothermal stability, dyeability, and soft texture, owing to the efficient penetration of chromium salts into skin fibers^10^. However, despite its desirable qualities, chrome tanning is widely condemned for its environmental impact, releasing trivalent chromium ions (Cr(III)) into water bodies^11^. The predominant disposal methods for chrome shavings, such as landfilling and incineration, carry significant risks of secondary pollution, particularly with hexavalent chromium, necessitating extensive and enduring remediation efforts^12^.

The concept of the circular economy offers a promising solution to the environmental challenges posed by the leather industry^13^. This concept has garnered significant attention from policy advocacy groups as a means to address sustainability issues^14^. This transformative approach advocates for a shift from the traditional linear model of production-consumption-disposal to a circular model, where waste is minimized through maximized reuse, and natural resource utilization is reduced^15^. Circular economy strategies applied to solid and liquid waste management in the leather industry hold the potential to significantly reduce waste quantities and facilitate the recovery of valuable materials like chromium^16^. Moreover, the circular economy concept not only offers environmental benefits but also proves to be more economical than the traditional economy model, as it reduces energy consumption and reliance on non-renewable resources^17^. By promoting the reuse of waste and lowering emissions of pollutants, this concept not only aligns with sustainability goals but also fosters the development of enterprises through the introduction of innovative technologies and enhancement of their goodwill^13^.

The biochemical decomposition of chromium leather wastes typically involves utilizing a gentle alkaline medium with specific enzymes as catalysts. Under these conditions, Chromium (III) hydroxide precipitates, forming a hydrated chromium cake with around 70% moisture content. This cake is then separated from hydrolyzed proteins through centrifugation or vacuum filtration. Despite containing residual protein substances, the hydrated chromium cake undergoes further purification before being reused in the tanning process. Additionally, the protein hydrolysate, which still contains chromium, can be utilized for the production of retaining or finishing agents in the leather industry^18^. Moreover, the characteristics of the residues from hydrolysis suggest that anaerobic digestion could serve as a potential alternative treatment method. Anaerobic processes offer advantages over aerobic methods, including reduced sludge production and energy consumption. Anaerobic digestion is a biochemical process that occurs in the absence of oxygen, facilitated by anaerobic microorganisms. This process converts complex organics into biogas, providing a sustainable approach for treating tannery wastewater^19^. This biogas can be efficiently utilized as a renewable energy source, powering leather industries and enhancing their waste management practices.

While adopting a circular approach can greatly improve environmental outcomes, it is essential to acknowledge that improper execution may lead to adverse environmental impacts and challenge the sustainability of producing value-added products within this system. Life Cycle Assessment (LCA) emerges as a pivotal decision-making tool in this context, regulated by ISO 14,040 and 14,044 standards since 2006^20^. LCA facilitates the identification, quantification, and evaluation of environmental impacts associated with a product, process, or activity. It assesses emissions into soil, water, and air, as well as resource extraction throughout the lifecycle, from raw material acquisition to waste disposal or valorization^20^. Widely utilized in research, LCA helps identify and mitigate environmental concerns in manufacturing systems while evaluating the environmental sustainability of alternative products^21^. Through comprehensive comparisons, LCA aids in making informed decisions to select sustainable alternatives for processes, products, services, and technologies^22^. By evaluating both positive and negative impacts, even those not immediately apparent, LCA assists decision-makers, policy-makers, and the public in understanding the role of waste valorization within a circular economy from an environmental perspective.

Despite the extensive application of LCA in evaluating environmental impacts in leather production, research specifically addressing chromium recovery from leather waste remains limited. For example, Kiliç et al.^23^ assessed the environmental impacts of tannery sludge treatment systems incorporating chromium recovery using LCA. They reported that the use of chemicals such as sulfuric acid, soda, and hydrogen peroxide during the chromium recovery process significantly contributed to environmental burdens, particularly in categories like acidification potential, abiotic depletion potential, and global warming potential. Additionally, they stated that incorporating anaerobic digestion into the treatment process effectively reduced sludge volume, energy use, and impacts on global warming potential, and abiotic depletion potential by over 30%. In another study, Rodríguez et al.^24^ assessed the environmental impacts of conventional wastewater treatment and chromium recovery from leather industry effluents based on LCA and using the ReCiPe method. Their analysis revealed that sludge disposal in conventional processes significantly contributed to environmental impacts due to high energy demand. In contrast, alternative treatments reduced CO_2_emissions by 1.5 kg/m² and environmental damage by 85%, primarily by decreasing energy consumption and minimizing waste. However, chemical usage in alternative processes was identified as a key contributor to impacts, suggesting potential areas for further optimization. Tasca and Puccini^25^ applied the LCA method to estimate the environmental impacts of retanning, fatliquoring, and dyeing processes in the leather industry while also considered chromium recovery from waste. They found that, despite substantial recovery and recycling of basic chromium sulfate, its production remains the primary contributor to metal depletion, specifically contributing 5.56E-02 kg Fe eq per kg of crust leather.

Chromium recovery from leather waste represents a paradigm shift in waste management, offering a beacon of hope in the fight against industrial pollution. By dissecting the environmental ramifications of biochemical decomposition and chromium reuse in tanning processes, the aim is to showcase the immense potential for reducing harmful emissions and conserving precious resources. Moreover, the exploration extends to the realm of biogas production, where anaerobic digestion emerges as a sustainable solution for managing tannery waste. Through meticulous LCA analysis, this study seeks to uncover the true environmental footprint of energy production and consumption from biogas, highlighting its capacity to slash greenhouse gas emissions and revolutionize energy consumption patterns. But the ambition doesn’t stop there. The study aspires to close the nutrient loop within the circular economy framework by examining the prospect of converting residues as fertilizer. In essence, this study is not just about research—it is about redefining the status quo, charting a course towards a cleaner, more sustainable future for the leather industry. Through innovative thinking, rigorous analysis, and unwavering commitment, it strives to catalyze positive change and inspire a new era of environmental stewardship in the industry. Another crucial aspect of this research is the exploration of opportunities and limitations of chromium recovery compared to the production of raw chromium, focusing on both environmental and energy perspectives—an area often overlooked in other studies. Table 1 compares the current study with other notable research studies on chromium recovery from leather waste.

**Table 1:** A comparison of the studies on chromium recovery for from leather waste.

Chromium recovery	Biogas production	Heat and electricity from biogas	Fertilizer production	Environmental impact assessment	Cumulative energy demand analysis	Refs
✓	✗	✗	✗	✓	✗	^23^
✗	✓	✗	✗	✗	✗	^26^
✓	✗	✗	✗	✓	✗	^27^
✓	✗	✗	✗	✗	✗	^28^
✓	✗	✗	✗	✗	✗	^29^
✗	✓	✗	✗	✗	✗	^30^
✓	✗	✗	✗	✗	✗	^31^
✗	✗	✗	✓	✗	✗	^32^
✓	✗	✗	✗	✗	✗	^33^
✓	✗	✗	✗	✓	✗	^25^
✗	✗	✗	✓	✗	✗	^34^
✓	✗	✗	✗	✗	✗	^33^
**✓**	**✓**	**✓**	**✓**	**✓**	**✓**	**Present Study**

## Methodology

### Chemical recycling of leather waste

**Fig. 1:**
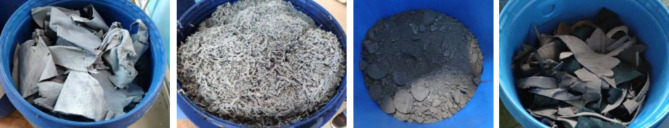
Leather waste samples, from left to right: Wet blue cuttings, wet blue shavings, dust, and finished tanned leather.

The leather waste used in this study consisted of wet blue leather residues obtained from local tanneries. It was collected representative samples of solid waste from Polish tanneries. These residues were primarily composed of chromium-tanned leather scraps. The shipments included cuttings and shavings of chromium-tanned leather (wet blue) with varying moisture contents, as well as dust from the processing and cuttings from finished tanned leathers, which are the primary solid waste materials generated in tanneries (Fig. 1). Notably, the samples with a low moisture content (< 20%) were processed as received. Wet samples, such as the wettest blue cuttings, were air-dried for preservation before further processing. Table 2 displays the characterization results of the various materials. The broader range of some values is due to differences between batches and shipments, as well as variations within the material from a single batch.

**Table 2:** Characterization of solid waste materials.

Parameter	Unit	Cuttings wet blue (various batches	Shavings wet blue	Dust	Cuttings finished tanned leathers (various batches)
Dry matter (delivery condition or air dried)	%	83.6–87.3	79.8–80.7	90.9–91.6	86.6–91.3
Organic dry matter	% dm	82.2–93.5	83.9–87.7	86.1–86.5	81.5–92.5
Total nitrogen	% dm	13.0–14.5	13.0–15.1	7.7–8.0	8.9–14.3
Raw fat	g/kg dm	2.0–37.3	6.2–33.5	69.0–69.7	30.5–65.8
Total chromium	% dm	2.88–3.86	2.74–3.30	2.25–23.38	2.66–4.39

The reagents used for hydrolysis included sodium hydroxide (NaOH), nitric acid (HNO_3_), sulfuric acid (H_2_SO_4_), hydrochloric acid (HCl), and phosphoric acid (H_3_PO_4_). Each reagent was prepared in various concentrations (0.5%, 1%, and 2%) to evaluate their effectiveness in leather disintegration and chromium separation. Initial hydrolysis experiments were conducted using a laboratory microwave system (Mars5plus, CEM), which allowed for parallel hydrolyses of different materials under controlled conditions. For each experiment, 3–7 g of leather waste were combined with 30–50 g of reagent in PTFE pressure vessels. The samples were heated from room temperature to 160 °C, over a 20-minute period, followed by a 60-minute hold time at 160 °C. After cooling, the hydrolysis samples were filtered for analysis.

**Fig. 2:**
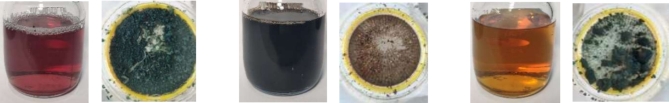
Liquid and solid phases of hydrolysis with water (left), nitric acid (middle), and sodium hydroxide (right).

Filtration was optimized to efficiently separate the solid and liquid phases post-hydrolysis. Several filtration methods were tested, including different types of filter papers (paper, glass) with varying retention sizes and filter areas, glass filter crucibles, pressure filtration, and vacuum filtration over different membranes. Vacuum filtration using a 0.45 μm polyether-sulfone membranes (PES) bottle-top filter was found to be the most effective for subsequent experiments. Hydrolysis experiments were conducted with water, sodium hydroxide, nitric acid, sulfuric acid, hydrochloric acid, and phosphoric acid, resulting in varied outcomes. Water produced a reddish liquid and greenish solid phase; nitric acid resulted in almost no solid phase, with a dark brown liquid phase; and sodium hydroxide yielded a yellow to amber liquid phase and a green precipitate as the solid phase (Fig. 2).

With the addition of sodium hydroxide, the leather disintegrated efficiently at temperatures between 140 °C and 160 °C, with 80–100% of the organic material transitioning to the liquid phase. At pH > 9, chromium precipitated out, resulting in more than 99% of chromium being transferred to the solid phase. This left the liquid phase nearly chromium-free, facilitating an efficient separation process.

**Fig. 3:**
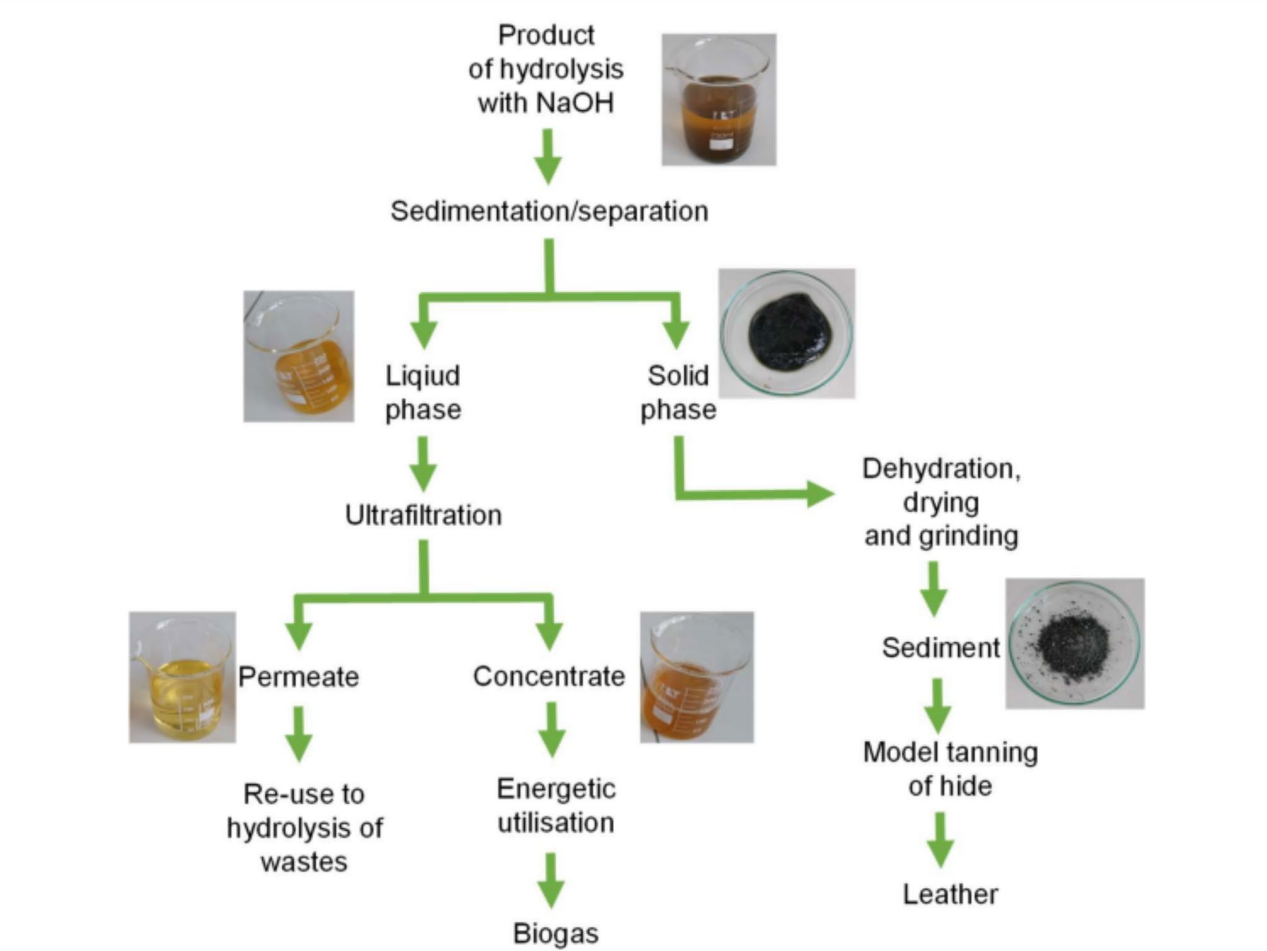
The process flow of leather waste treatment, chromium recovery, and biogas utilization for energy production.

The chromium-free liquid fractions from the thermo-chemical treatment were utilized as feedstock for biogas production. Anaerobic digestion was performed, degrading about 70% of the organic content in the leather residues. The initial heat treatment enhanced the specific biogas potential of the leather residues by over ten times compared to untreated anaerobic conditions. Under optimal conditions, one ton of wet blue residue produced approximately 120 cubic meters of methane. The digestate resulting from anaerobic digestion, containing high concentrations of readily accessible nitrogen, was evaluated for its potential use as an agricultural fertilizer. The nutrient content and environmental impacts of using this digestate as fertilizer were analyzed. The filtrates and solid residues were analyzed for chromium content, organic material degradation, and biogas production potential using standard analytical techniques such as spectrophotometry and gas chromatography. Figure 3 illustrates the detailed process steps involved in leather waste treatment as outlined in this study, showcasing the journey from hydrolysis to chromium recovery and the utilization of biogas for energy production.

### Life cycle assessment methodology

LCA, also known as life cycle assessment, is a critical and comprehensive quantitative approach for evaluating the sustainability of human activities. Recognized as a leading accounting tool, LCA meticulously tracks all resource inputs, including energy and materials, throughout the lifecycle of a product or service^35^. This method is instrumental in identifying areas for improvement to minimize environmental impacts and enhance sustainability. By enabling the comparison of different alternatives, LCA supports informed decision-making and promotes sustainable practices^36^. In this study, the environmental evaluation of the proposed scenario is conducted in accordance with LCA principles, specifically following ISO 14,040 and 14,044 standards^37,38^.

The standard LCA methodology consists of four interconnected steps: goal and scope definition, life cycle inventory (LCI), life cycle impact assessment (LCIA), and interpretation. These phases are described in detail as follows. The goal and scope definition phase establish the objectives and boundaries of the LCA, specifying the purpose of the study, the system to be analyzed, and the functional unit to measure the environmental performance^39^. In the life cycle inventory (LCI) phase, data on all the inputs and outputs associated with the product or service lifecycle are collected and quantified^40^. This includes raw material extraction, energy consumption, emissions, and waste generation. The life cycle impact assessment (LCIA) phase evaluates the data gathered in the LCI phase to understand the potential environmental impacts, categorizing and quantifying the effects on various environmental indicators, such as global warming potential, resource depletion, and ecotoxicity^41^. The final phase, interpretation, involves analyzing the results from the LCIA to draw conclusions and make recommendations, aiming to identify significant environmental impacts, assess the reliability of the results, and propose strategies for improvement. By adhering to these structured phases, this study leverages LCA to critically assess and enhance the environmental sustainability of the proposed scenario.

**Fig. 4:**
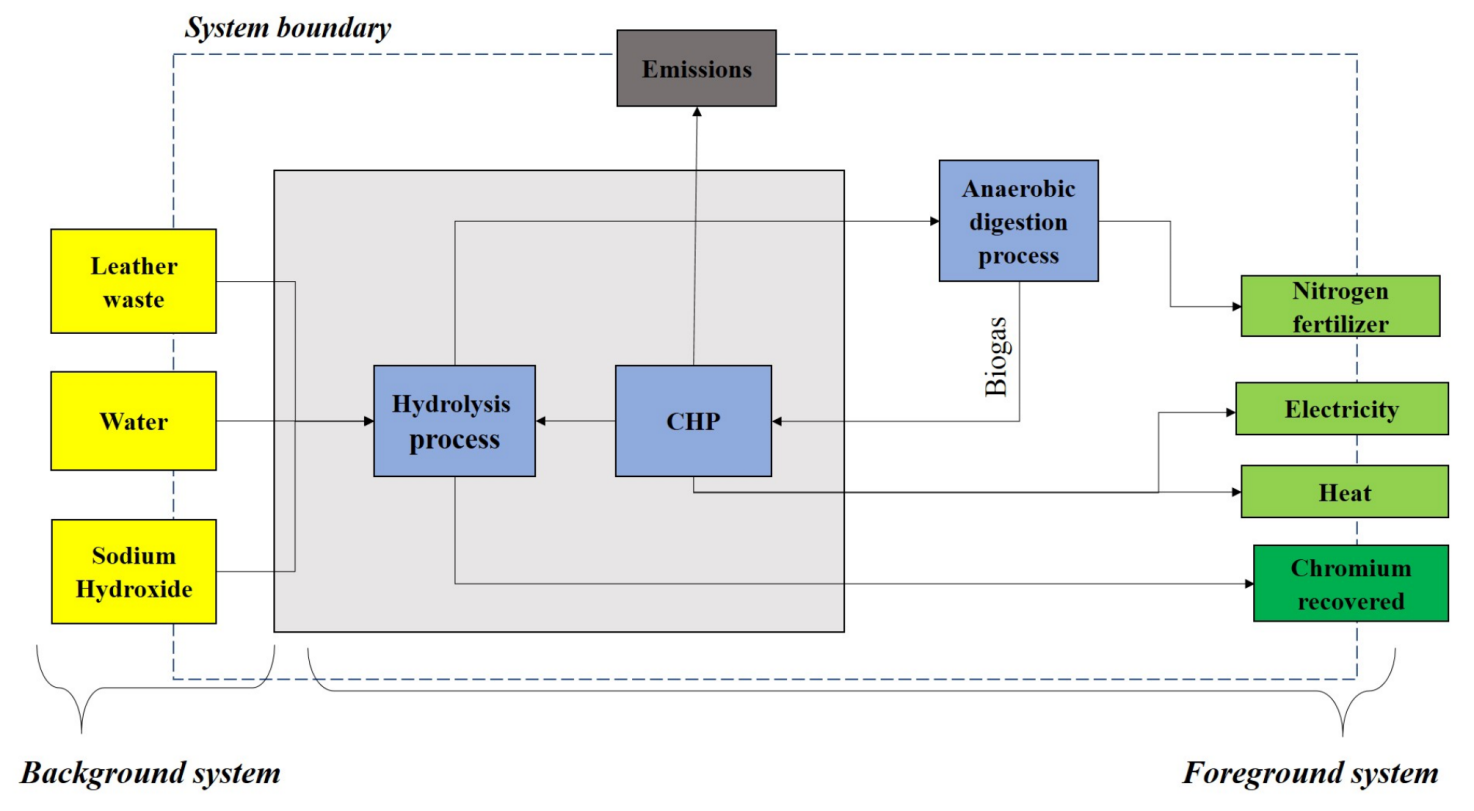
System boundary for chromium recovery from leather waste in the current study.

The goal and scope definition is a crucial first step in any LCA study, as it significantly influences the subsequent steps. The primary objective of this study is to evaluate the environmental impacts of chromium recovery from leather waste, the combined heat and power (CHP) unit’s generation of heat and electricity from biogas, and the recovery of nitrogen for use as fertilizer. This assessment aims to identify the environmental limitations and opportunities associated with implementing a circular economy approach in leather waste management. The analysis results will provide valuable insights to policymakers and stakeholders, facilitating informed decisions about the most sustainable and environmentally responsible options. To achieve these objectives, a detailed and precise description of the system under investigation is essential. This includes defining its function and boundaries, as these aspects are foundational to the methodology employed in subsequent steps. In this study, the functional unit (FU) is defined as 1 kg of chromium recovered from leather waste, which serves as a basis for quantifying the system’s function and enabling the comparison of results. Additionally, defining the system boundary is vital to the study. The system boundary delineates the processes included in the analysis, specifically focusing on chromium recovery, fertilizer production, and energy generation from leather waste (Fig. 4). This clear delineation ensures that all relevant processes are considered and accurately assessed within the study’s scope.

It should be noted that allocation proves to be a robust strategy in an attributional LCA, especially when aiming to identify production-level focal points accurately. In this study, we adopted an allocation method rooted in the economic worth of all relevant co-products, a necessity due to the multiple co-products involved. Chromium emerges as the primary product, constituting 38% of the total, followed by electricity, heat, and nitrogen fertilizer at 17%, 21%, and 24%, respectively. This method ensures a comprehensive assessment, shedding light on the nuanced contributions of each component in the production process.

The inventory phase is a pivotal component of LCA studies as it involves the systematic collection and organization of data, which is essential for ensuring the accuracy and validity of the analysis^42^. This phase typically utilizes two types of data: background and foreground. Background data, sourced from the EcoInvent database (version 3) available in SimaPro, includes information on the environmental impacts associated with the production and distribution of materials and energy carriers used in the chromium recovery process from leather waste. Foreground data, on the other hand, is specific to the study and involves detailed records of the quantity and type of materials and energy resources consumed, as well as the outputs generated from experiments conducted at PFI Germany. Table 3 provides a comprehensive overview of the inventory data utilized in this study, ensuring a thorough and reliable foundation for subsequent phases of the LCA.

**Table 3:** Inputs and outputs referred to 1 kg of chromium recovery from leather waste treatment.

Items	Component	Unit	Amount
Input	Leather waste	kg	31.51
	Water	kg	122.27
	Sodium hydroxide	kg	3.78
Intermediate product	Biogas	kWh	38.87
Output	Chromium	kg	1
	Nitrogen fertilizer	kg	2.60
	Electricity	kWh	14.70
	Heat	kWh	10.50

For conducting the LCA, this study follows several key assumptions. One of the main assumptions is that leather waste is considered a zero burden. It is also assumed that the biogas produced is used as fuel in CHP system, with the heat generated being directed to the hydrolysis process (thermal efficiency: 40%; electricity efficiency: 45%). Due to the unavailability of emission data from the combustion of the produced biogas in this study, it is assumed that these emissions are similar to those from other biogases, and emissions were estimated using data from SimaPro software database instead of direct measurements. Emissions from the anaerobic digestion process are considered zero. Additionally, it is important to note that the CHP section did not physically exist at the laboratory scale and was instead hypothetically simulated and modeled for the purposes of this study. In the hydrolysis section, employing a heat exchanger with a 70% efficiency significantly reduces the system’s additional heating requirements by recovering and reusing heat within the process. This efficiency level indicates that 70% of the heat from the outgoing stream is transferred to the incoming stream, thereby decreasing the energy needed for heating. Additionally, 10% of the generated electricity is utilized to energy requirements of lab.

The study also has several limitations in the LCA. It did not account for the environmental impact of transporting waste and chemicals to the laboratory, as accurate data for this were unavailable. Furthermore, the environmental impacts associated with the production of the CHP unit were not calculated. This limitation also applies to other equipment involved in biogas production, such as the digester, as data was not available. Additionally, the environmental impacts of the laboratory building were not included in the analysis.

While the inventory phase provides a detailed list of substances with potential environmental impacts, relying solely on this information for decision-making is insufficient^43^. Therefore, the LCIA is implemented. The LCIA step is critical for determining the magnitude and significance of the environmental impacts associated with a product or service. It uses the data collected during the LCI phase to identify and evaluate environmental burdens. Detailed guidelines for the LCIA step are provided by ISO 14,040 ^37^. In this study, SimaPro software (v.9) is employed to model and assess the inventory data, utilizing the ReCiPe (H) V1.13 method, which considers 18 midpoints and three damage categories for comparison^44^. This approach allows for a comprehensive evaluation of the environmental impacts, aiding in more informed decision-making and policy development. Indeed, the ReCiPe impact assessment method was selected due to its comprehensive capabilities and broad applicability, as reported by Aghbashlo et al.^45^. They emphasized that ReCiPe covers more than 3000 substances, making it more extensive than other methods. ReCiPe also offers a wide range of midpoint impact categories, addressing various environmental concerns. Additionally, ReCiPe allows for the presentation of environmental impacts in both midpoint and endpoint level and at a global scale, in contrast to methods like CML that only focuses on midpoints and Europe. It can also provide weighted environmental impact results. This flexibility, combined with its ability to evaluate environmental impacts from multiple perspectives (Egalitarian, Hierarchist, and Individualist), provides a robust framework for addressing subjective ambiguities and ensuring thorough, balanced assessments of environmental impacts.

It should be noted that environmental impacts in midpoint level offer valuable insights into the environmental effects of unfavorable emissions and resource depletion, enabling environmental experts to identify specific areas for improvement and develop targeted interventions^46,47^. However, these categories can be challenging for the public to fully comprehend^36^. In contrast, environmental impacts in endpoint levels simplify the analysis, making the environmental impacts more accessible for policymakers and the general public, thus supporting effective policy-making and decision-making^47^. Therefore, the current study incorporates both midpoint and endpoint categories in its environmental assessment using LCA to provide a comprehensive view of the environmental burdens.

The current study also evaluates the energy cumulative demand (CED) of chromium recovery, providing critical insights into energy resource utilization. Using the advanced SimaPro software, this assessment quantifies the potential depletion of energy resources, underscoring the importance of energy efficiency in the chromium recovery process from leather tanning residues.

## Results and discussion

### Material testing of the tanned leather

In order to know the technical performance of recovered chrome for reuse in the leather industry, the physical and chemical parameters of leather tanned with the use of chromium recovered from waste were investigated. The test results were then compared with the physico-chemical parameters of leathers traditionally tanned with a commercially available chrome tanning agent. The thickness, tensile strength, elongation, tear strength, burst height, finish adhesions, chromium and moisture content were analyzed. Based on the results of physical and chemical tests, it was found that the physical and chemical characteristics of leathers tanned with the addition of chromium recovered from waste are similar to those of traditionally tanned leather (Table 4).

**Table 4:** Parameters of leather tanned using of chromium recovered from waste in the form of a concentrate separated from the alkaline hydrolysate.

Parameter	Leather tanned with a commercial tannin	Leather tanned using chromium recovered from waste
Thickness [mm]	1.27	1.27
Tensile strength [N/mm^2^]	22.64	21.86
Elongation [%]	50	47
Tearing force [N]	92.83	91.38
Burst height (Lastometr)	9.9	9.5
Adhesion of the finish [N/cm]	4.2	5.4
Chromium(III) content converted to Cr_2_O_3_ [%]	4.33	4.18
Moisture content [%]	12.2	11.7

**Fig. 5:**
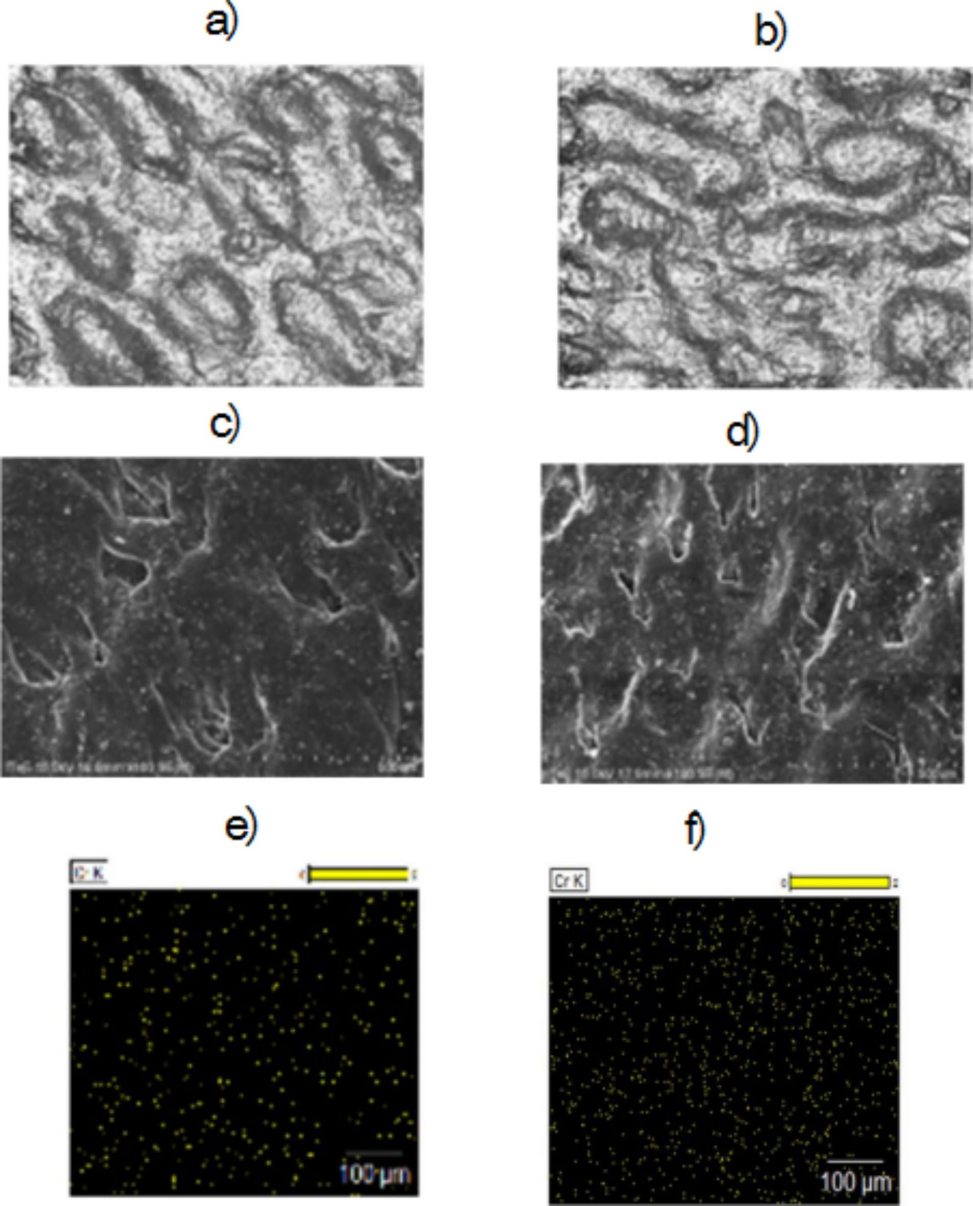
Images of leather surfaces made with a 3D microscope: (**a**) traditionally tanned leather, (**b**) leather tanned with the use of chromium recovered from hydrolysed waste alkaline; Images of leather surfaces made with SEM (over 100x): (**c**) traditionally tanned leather, (**d**) - leather tanned with the use of chromium recovered from hydrolysed waste alkaline; EDS maps of surface distribution of chromium: (**a**) traditionally tanned leather, (**b**) leather tanned with the use of chromium recovered from alkaline hydrolysed waste.

Samples of leathers tanned with the use of chromium recovered from waste were subjected to microscopic observations which compared them with microscopic images of leathers tanned conventionally with a commercially available chrome tanning agent. A 3D microscope and a scanning electron microscope were used in the research. Pictures of traditionally tanned leather and with the use of chromium addition recovered from waste in the form of a concentrate or sediment are presented in Fig. 5a, b, c, and d, respectively. On the basis of microscopic observations, it was found that the use of chromium recovered from waste had no negative effect on the structure of the skin surface. Moreover, the distribution of chromium on the surface of leather tanned with the use of chromium recovered from waste was even and similar to the distribution of chromium on the surface of traditionally tanned leather (Fig. 5e, f).

The nitrogen fertilizer underwent testing for heavy metal content to assess its suitability for direct use or the necessity of additional purification for plant applications. Table 5 presents the heavy metal content of the nitrogen fertilizer produced in this study. The analysis reveals trace amounts of various heavy metals, with arsenic (As), lead (Pb), cadmium (Cd), and zinc (Zn) all being present in levels below detection limits of 0.1, 0.01, 0.005, and 0.005 mg/kg, respectively. Mercury (Hg) was detected at a concentration of less than 0.5 mg/kg, while silver (Ag) was found at 0.03 mg/kg. The highest concentration was observed for copper (Cu), at 3.65 mg/kg. These findings suggest that while the levels of most heavy metals are relatively low, the presence of Cu and Ag, in particular, indicates that further purification is necessary to ensure the fertilizer’s safety for direct plant use. Kumar et al.^48^ reported that excessive Cu can be toxic to plants and humans, as it disrupts plant growth, nutrient uptake, and poses health risks through contaminated crops. Natalia et al.^49^ also claimed that higher the concentration of Ag in the soil, the more significant the toxic effect on the biological indicators of soils such as catalase and dehydrogenases activity, the number of bacteria, length of root, etc.

**Table 5:** Heavy metal content in nitrogen fertilizer produced in this study.

Name of heavy metals	As	Pb	Cd	Zn	Hg	Ag	Cu
Content in nitrogen fertilizer (mg/kg)	< 0.1	< 0.01	< 0.005	< 0.005	< 0.5	0.03	3.65

Table 6also presents key parameters of biogas production, offering valuable insights into the efficiency of biogas generation from organic dry matter. The specific biogas yield of 0.492 m³/kg organic dry matter reflects the overall gas output, while the methane content of 58.2% highlights the energy-rich component of the biogas. This figure is in the range of 270–710 m³/kg organic dry matter reported by a large number of studies around biogas potential from organic substrates^50,51^. Additionally, the specific methane yield of 0.286 m³/kg organic dry matter demonstrates the amount of methane produced per unit of organic matter. These findings underscore the effectiveness of anaerobic digestion as a sustainable waste-to-energy solution for leather industry, reinforcing its viability in managing organic waste while producing valuable energy.

**Table 6:** Key parameters of biogas production in current research.

Parameter	Value
Specific biogas yield	0.492 m^3^/kg organic dry matter
Methane content	58.2%
Specific methane yield	0.286 m^3^/kg organic dry matter

### Environmental impact of chromium recovery from leather tanning residues

In Table 7, the environmental impacts of the recovery of per kg of chromium from leather tanning residues are presented. The impacts are categorized based on impact categories, highlighting the total impact and the contributions from sodium hydroxide, tap water, and biogas combustion. Based on Table 7, the total impact on fossil depletion amounts to $7.39E-02 per FU that major contributor to this category is sodium hydroxide, accounting for 98.36% of the total impact on fossil depletion impact category. The total impact on metal depletion is $1.03E-02 per FU, with sodium hydroxide again being the largest contributor at 92.33%. This indicates a significant reliance on fossil and metal resources during the sodium hydroxide production used in chromium recovery process.

Per kg of chromium recovery from leather tanning residues also leads to an impact of 2.43E-06 DALY on climate change, human health impact category. Sodium hydroxide contributes 96.92% to this category. In addition, chromium recovery based on method provide in this study results in 1.25E-06 DALY/FU impact on particulate matter formation that sodium hydroxide contributes 94.80% to this category. Similar to particulate matter formation, sodium hydroxide plays a critical role on photochemical oxidant formation caused by chromium recovery from leather tanning residues. The total impact on photochemical oxidant formation is reported 2.35E-10 DALY/FU, with sodium hydroxide contributing 90.96%. The total impact on human toxicity is calculated at 5.95E-07 DALY/FU, with sodium hydroxide contributing 96.91%. According to Table 7, the total impact on ionizing radiation impact category is also reported 3.79E-09 DALY/FU. The major contributor, sodium hydroxide, accounts for 97.03%. The total impact on ozone depletion is 3.16E-09 DALY per kg of chromium recovery from leather tanning residues, with sodium hydroxide contributing an overwhelming 99.85%. These impacts are all related to the human health damage category, highlighting the critical role that sodium hydroxide plays in the environmental footprint of chromium recovery from leather tanning residues. The findings underscore the need to consider the environmental burdens associated with chemical inputs, particularly sodium hydroxide, in the development and optimization of more sustainable chromium recovery processes.

Based on Table 7, sodium hydroxide emerges as a major contributor to multiple impact categories associated with ecosystem damage category. Its substantial influence underscores the critical need to address its environmental footprint within the chromium recovery process. In general, the total impact on climate change, ecosystems is 1.37E-08 species.yr/FU, with sodium hydroxide responsible for 96.92% of this impact. The impact on agricultural land occupation is also 1.05E-09 species.yr/FU, with sodium hydroxide accounting for 97.91%, indicating extensive land use impacts from its production. For urban land occupation impact category caused by chromium recovery process, the impact is calculated 5.63E-10 species.yr/FU, with sodium hydroxide contributing 98.42%, signifying considerable urban land use and habitat disruption.

In terms of natural land transformation, chromium recovery from leather tanning residues leads to an impact of 4.94E-10 species.yr/FU, with sodium hydroxide being the largest contributor at 98.14%. The impact on freshwater ecotoxicity is also 5.32E-11 species.yr/FU, with sodium hydroxide accounting for 96.80%, indicating substantial toxic effects on freshwater ecosystems. The impact on terrestrial acidification is 5.44E-11 species.yr/FU, with sodium hydroxide contributing 90.61%. Based on Table 7, freshwater eutrophication impact category for per kg chromium recovered is 3.73E-11 species.yr, with sodium hydroxide accounting for 97.71%. The impact on terrestrial ecotoxicity is also 2.86E-11 species.yr/FU, with sodium hydroxide being the primary contributor at 97.85%. Lastly, the impact of chromium recovery from leather tanning residues on marine ecotoxicity is 9.89E-12 species.yr/FU, with sodium hydroxide contributing 96.81%. Overall, sodium hydroxide is identified as the predominant contributor to environmental impacts across various categories in the chromium recovery process. These findings emphasize the need for careful consideration and potential mitigation strategies for the use of sodium hydroxide in order to improve the environmental sustainability of chromium recovery from leather tanning residues. In the study of Kiliç et al.^23^, it is reported that the use of sodium hydroxide in chromium recovery from leather tanning residues contributes significantly to environmental impacts, particularly in categories such as acidification, abiotic depletion, and global warming potential. However, as reported in literature review, the results are inherently different from the methodology used in this study. Therefore, exact comparison of the results is not sensible.

**Table 7:** Environmental impact assessment of chromium recovery from leather tanning residues using various impact categories associated with the ReCiPe method (FU = 1 kg of chromium recovered).

Impact category	Unit	Total	Sodium hydroxide	Tap water	Energy production from biogas
Climate change Human Health	DALY	2.43E-06	2.35E-06	2.11E-08	5.37E-08
Ozone depletion	DALY	3.16E-09	3.15E-09	2.88E-12	1.95E-12
Human toxicity	DALY	5.95E-07	5.76E-07	7.81E-09	1.06E-08
Photochemical oxidant formation	DALY	2.35E-10	2.14E-10	1.95E-12	1.93E-11
Particulate matter formation	DALY	1.25E-06	1.18E-06	9.01E-09	5.59E-08
Ionizing radiation	DALY	3.79E-09	3.68E-09	9.06E-11	2.18E-11
Climate change Ecosystems	species.yr	1.37E-08	1.33E-08	1.19E-10	3.04E-10
Terrestrial acidification	species.yr	5.44E-11	4.93E-11	3.83E-13	4.73E-12
Freshwater eutrophication	species.yr	3.73E-11	3.65E-11	4.97E-13	3.60E-13
Terrestrial ecotoxicity	species.yr	2.86E-11	2.80E-11	2.96E-13	3.18E-13
Freshwater ecotoxicity	species.yr	5.32E-11	5.15E-11	3.75E-13	1.33E-12
Marine ecotoxicity	species.yr	9.89E-12	9.58E-12	7.18E-14	2.44E-13
Agricultural land occupation	species.yr	1.05E-09	1.03E-09	1.09E-11	1.11E-11
Urban land occupation	species.yr	5.63E-10	5.54E-10	4.29E-12	4.63E-12
Natural land transformation	species.yr	4.94E-10	4.84E-10	3.27E-12	5.91E-12
Metal depletion	$	1.03E-02	9.49E-03	1.94E-04	5.94E-04
Fossil depletion	$	7.39E-02	7.27E-02	6.71E-04	5.42E-04

This research delves into the environmental impacts of chromium recovery from leather tanning residues, focusing on endpoints within damage categories. By quantifying the potential damage caused to various environmental endpoints, such as resources, human health, and ecosystems, this assessment provides valuable insights into the overall environmental performance of the system under study. The valorization of leather tanning residues for chromium recovery results in a damage of $8.42E-02 per FU to resources. Furthermore, the damage to human health and ecosystems is measured at 4.28E-06 DALY per FU and 1.60E-08 species.yr per FU, respectively. It should be noted that sodium hydroxide plays a primary role in these damages.

**Fig. 6:**
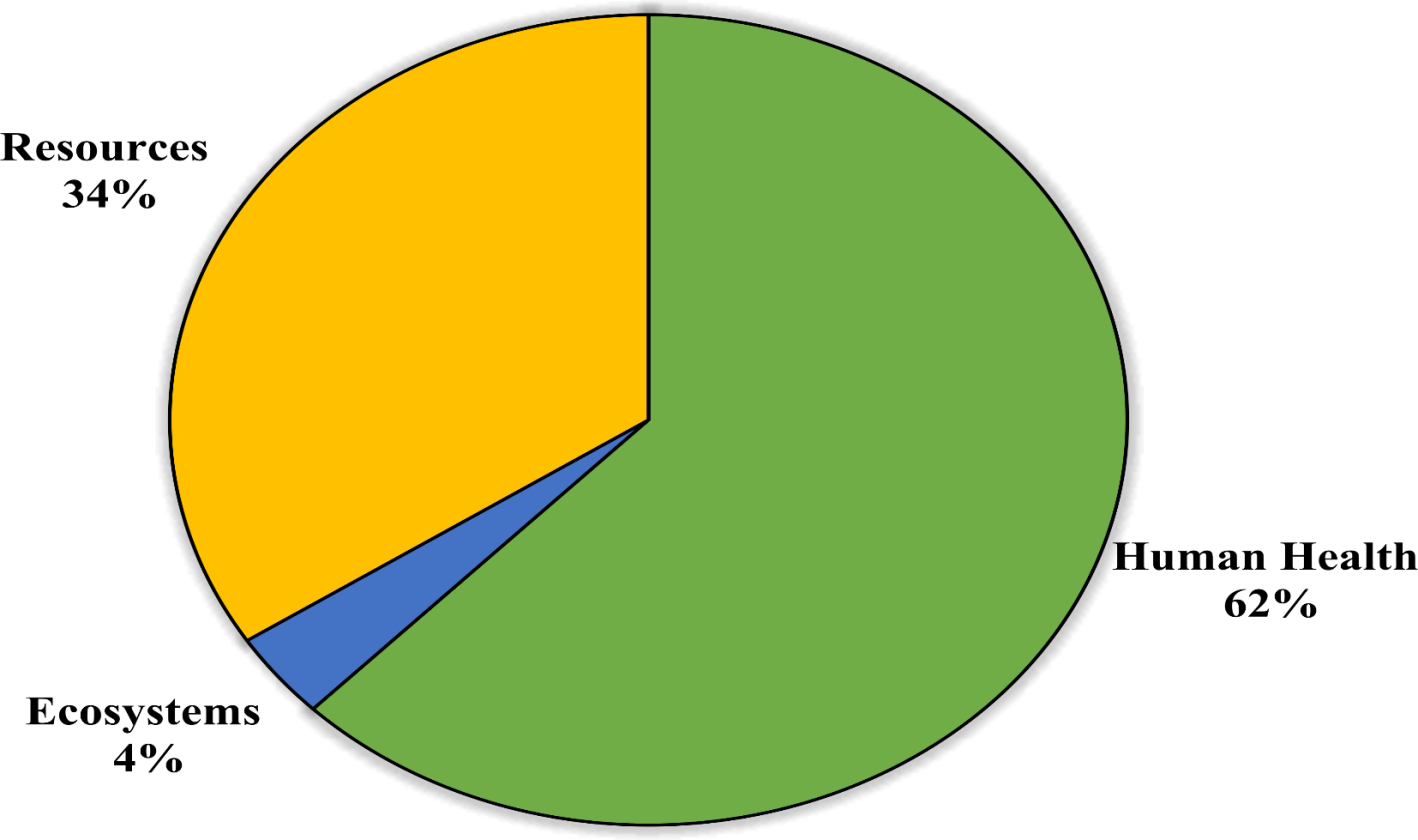
Share of damage categories in total environmental impact of chromium recovery from leather tanning residues.

In the culmination of the LCA, a crucial optional step—weighing—was conducted to comprehensively evaluate the environmental impact of recovered chromium. The findings revealed that the total environmental impact of chromium recovery stands at 201.04 mPt/FU. The visualization in Fig. 6 vividly illustrates these disparities, highlighting the disproportionate burden placed on human health. With a remarkable 62% share, the impact on human health takes center stage, signaling the urgent need for mitigative measures to protect public well-being. Additionally, resources emerge as a significant consideration, accounting for 34% of all environmental damages. This underscores the intricate relationship between industrial processes and resource utilization, emphasizing the imperative of adopting sustainable practices to minimize environmental degradation. Conversely, the ecosystems’ endpoint, with a mere 4% share, exhibits the least impact on the environment in this process. While this percentage is comparatively small, it emphasizes the importance of safeguarding ecosystems from industrial activities.

It should be noted that when the recovery of a compound is environmentally sustainable, it exhibits a lower environmental impact compared to its production from raw sources. To assess the environmental opportunities and limitations of chromium recovery from leather factory residues, its environmental impacts have been investigated and compared with those of raw chromium production (based on EcoInvent database: Chromium {GLO}| market for | APOS, U). The results are presented in Table 8. The comparison between chromium recovery from leather tanning residues and the production of raw chromium reveals substantial environmental benefits, as reflected in the percentage changes across various impact categories. The recovery process shows a significant reduction of 93.57% in climate change, human health impact category. Human toxicity impacts decrease by 90.23%, highlighting the safety of the recovery process. Additionally, there are notable reductions of 92.17% and 92.90% in photochemical oxidant formation and particulate matter formation, respectively, emphasizing improved air quality. The impact on ionizing radiation is reduced by 92.39%. Impact categories related to ecosystems also show significant decreases, with climate change, ecosystems impact category reduced by 93.60%, and other categories associated with ecosystems also demonstrating reductions between 87.21% and 92.75%. Furthermore, resource depletion impacts see dramatic reductions, with metal depletion decreasing by 99.52% and fossil depletion by 93.84%. These substantial percentage reductions underscore the environmental advantages of chromium recovery, though attention to reducing ozone depletion impacts remains necessary.

Based on Notarnicola et al.^52^, basic chromium sulfate is identified as the key impacting chemical at the tannery level, affecting all impact categories in both systems analyzed. Also, Yang et al.^53^ reported that the mining process of chromium powder emits substantial harmful substances, such as carbon dioxide, leading to the unsustainability of leather production. This highlights the environmental challenges associated with chromium mining and underscores the importance of adopting more sustainable practices in the leather industry. In another study, Kılıç et al.^54^ reported that tannery operations consume 25.3 MJ of energy, 126.17 L of water, and 2.83 kg of chemicals, emitting 8.54 kg CO_2_ eq. to produce 1 m² of finished shoe leather. Tanning, retaining, and finishing are the most environmentally burdensome phases due to their heavy chemical use. In particular, the production of chemicals like chromium oxide significantly contributes to greenhouse gas emissions, impacting climate change.

Thus, the recovery of chromium not only reduces its discharge into the environment but also significantly decreases the environmental impacts of leather production when the recovered chromium is reused in the industry. This underscores the dual benefit of chromium recovery in minimizing environmental pollution and enhancing the sustainability of leather manufacturing processes.

**Table 8:** Comparative environmental impact of recovered chromium vs. raw chromium production.

Impact category	Unit	Chromium recovered	Raw chromium	% Change
Climate Change, Human Health	DALY	2.43E-06	3.78E-05	−93.57%
Ozone depletion	DALY	3.16E-09	3.46E-09	−8.67%
Human toxicity	DALY	5.95E-07	6.09E-06	−90.23%
Photochemical oxidant formation	DALY	2.35E-10	3.00E-09	−92.17%
Particulate matter formation	DALY	1.25E-06	1.76E-05	−92.90%
Ionizing radiation	DALY	3.79E-09	4.98E-08	−92.39%
Climate change, Ecosystems	species.yr	1.37E-08	2.14E-07	−93.60%
Terrestrial acidification	species.yr	5.44E-11	6.30E-10	−91.37%
Freshwater eutrophication	species.yr	3.73E-11	4.63E-10	−91.94%
Terrestrial ecotoxicity	species.yr	2.86E-11	2.65E-10	−89.21%
Freshwater ecotoxicity	species.yr	5.32E-11	4.16E-10	−87.21%
Marine ecotoxicity	species.yr	9.89E-12	7.88E-11	−87.45%
Agricultural land occupation	species.yr	1.05E-09	9.26E-09	−88.66%
Urban land occupation	species.yr	5.63E-10	5.87E-09	−90.41%
Natural land transformation	species.yr	4.94E-10	6.81E-09	−92.75%
Metal depletion	$	1.03E-02	2.13E + 00	−99.52%
Fossil depletion	$	7.39E-02	1.20E + 00	−93.84%

**Fig. 7:**
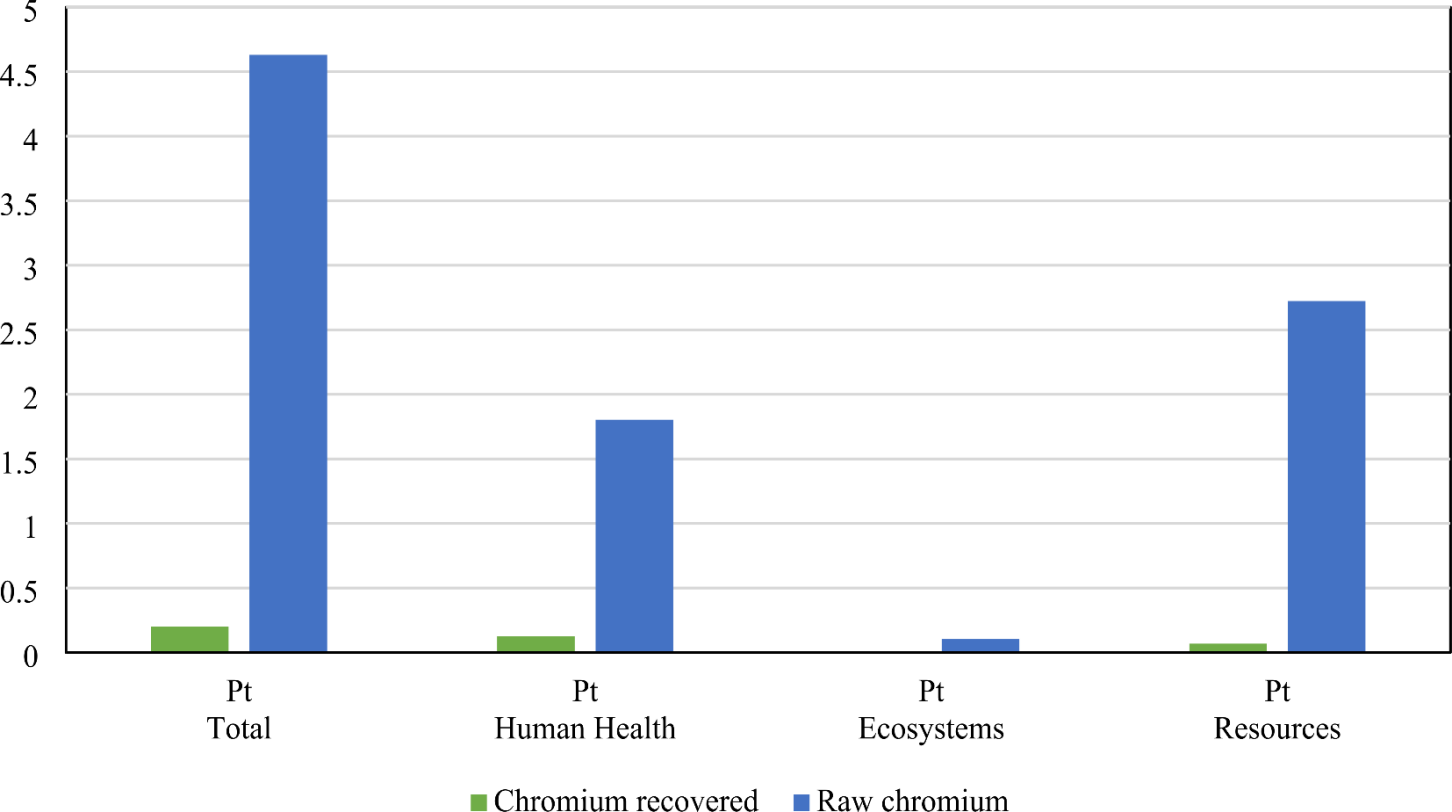
Comparative weighted environmental impact of recovered chromium vs. raw chromium production.

The comparison between the environmental impacts of recovered chromium from leather tanning residues and raw chromium production reveals significant benefits of the recovery process, as demonstrated by the substantial percentage changes across different damage categories. The weighted total environmental damage category of recovered chromium is dramatically lower, with a reduction of 95.65% (0.20 Pt/FU compared to 4.63 Pt/FU), as shown in Fig. 7. Weighted human health damage category see an impressive decrease of 93.05% (0.12 Pt/FU compared to 1.80 Pt/FU), emphasizing the reduced harm to human health associated with the recovery process. The weighted damage on ecosystems is reduced by 93.25% (0.006 Pt/FU vs. 0.103 Pt/FU), highlighting the recovery process’s lesser impact on biodiversity and natural habitats. Additionally, the impact on resources shows a significant reduction of 97.47% (0.068 Pt/FU vs. 2.72 Pt/FU), underscoring the efficiency and sustainability of using recovered chromium over raw chromium. These substantial reductions across all damage categories underscore the environmental advantages of chromium recovery from leather tanning residues, highlighting its potential for mitigating environmental degradation and promoting sustainable resource utilization. According to Chowdhury et al.^55^, the release of chromium significantly impacts both ecosystem health and human health. Processes such as tanning, re-chroming, neutralization, and acid washing contribute to elevated human toxicity due to the high concentration of total chromium in contaminated water, surpassing acceptable limits. Therefore, strategies such as chromium recovery, reuse, or chemical modification of chromium tanning salts are recommended to mitigate these adverse effects and improve environmental sustainability.

**Fig. 8:**
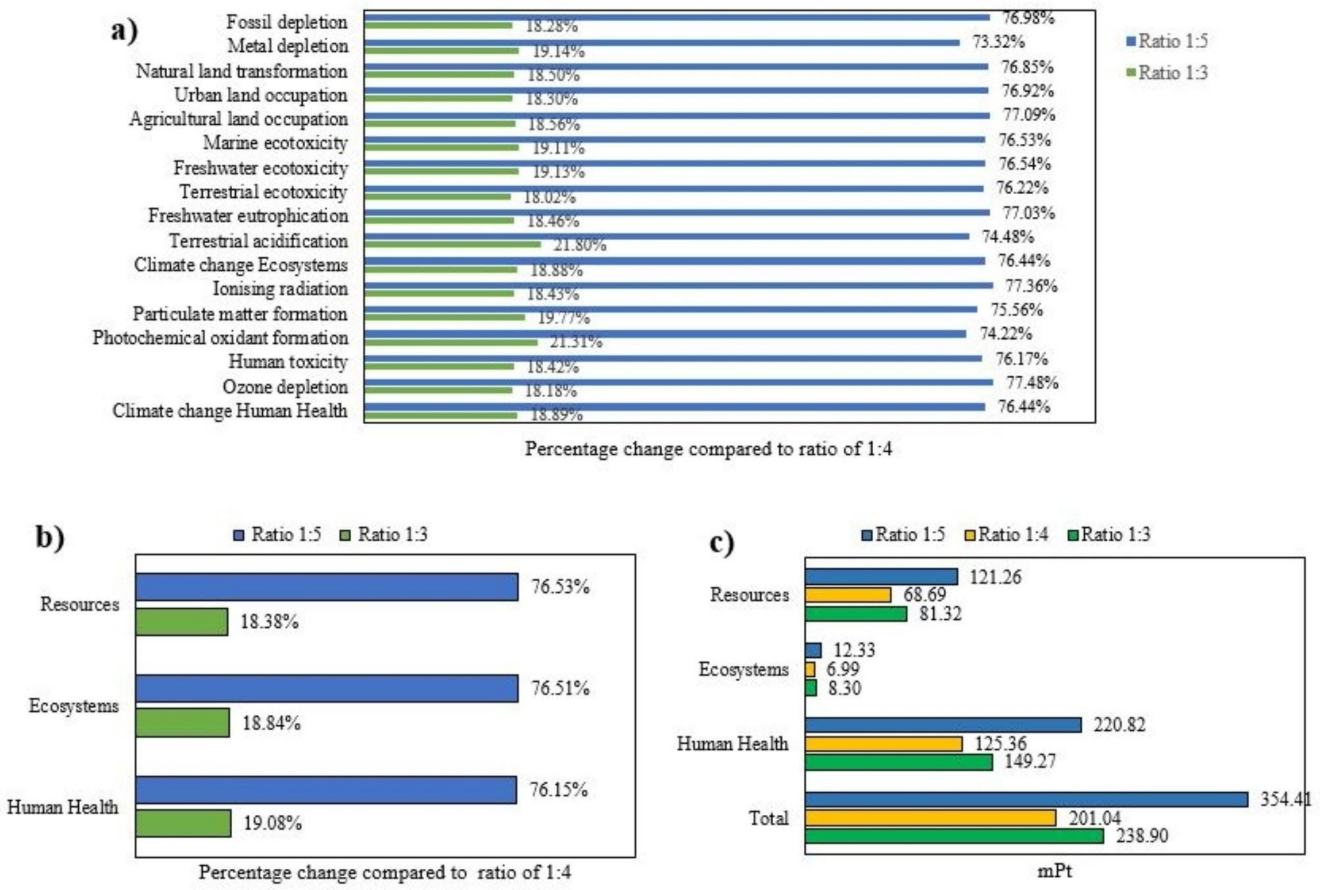
Sensitivity analysis of environmental impacts for chromium recovery: comparative assessment of 1:3, 1:4, and 1:5 ratios across (**a**) midpoint, (**b**) endpoint, and weighted endpoint categories.

The environmental impact of chromium recovery from leather waste is significantly influenced by the quantity of sodium hydroxide used during the process. To examine the effects of varying sodium hydroxide concentrations on recovery efficiency per kilogram of chromium, a sensitivity analysis was performed. The baseline experiment was conducted at a ratio of 1:4 (300 g leather and 1,164 g sodium hydroxide solution, including 36 g of sodium hydroxide) with a concentration of 3%. For sensitivity analysis, additional experiments were carried out using ratios of 1:3 and 1:5. The 1:5 ratio exhibited improved separation efficiency and demonstrated potential for industrial scalability, despite requiring higher energy input. On the other hand, the 1:3 ratio encountered practical separation challenges under laboratory conditions, though these issues could be addressed through appropriate technological interventions during upscaling. The results of the sensitivity analysis, showing the environmental impacts for each kilogram of chromium recovery across midpoint, endpoint, and weighted endpoint categories, are illustrated in Fig. 8.

Based on the results of the sensitivity analysis, the environmental impacts of both the 1:3 and 1:5 ratios, compared to the baseline ratio of 1:4, show an upward trend across midpoint, endpoint, and weighted endpoint categories. The 1:5 ratio, in particular, exhibits a substantial increase, with environmental impacts rising by nearly 80%. In contrast, the 1:3 ratio demonstrates a relatively moderate increase, with impacts escalating by approximately 20%. These results underscore the importance of sensitivity analysis as a tool to guide process optimization. It not only identifies potential environmental burdens but also aids in designing strategies for achieving an optimal balance between operational efficiency and environmental sustainability, particularly during industrial upscaling. This approach ensures that decision-makers have a comprehensive understanding of the implications of process variations, enabling more informed and sustainable choices in chromium recovery technologies.

### Energy analysis based on cumulative energy demand

The energy assessment of chromium recovery from leather tanning residues is conducted using various impact categories associated with the CED approach. The findings, detailed in Table 9, highlight the distribution of energy consumption across different sources and processes. The total energy demand for recovering 1 kg of chromium is 2.61E + 01 MJ. Sodium hydroxide is the primary contributor, accounting for 2.56E + 01 MJ, which is the bulk of the total energy consumed. This underscores the significance of sodium hydroxide in the energy footprint of the chromium recovery process. The energy assessment reveals that sodium hydroxide is the dominant contributor to the total energy demand across all categories, highlighting the need for strategies to reduce its consumption or find more energy-efficient alternatives.

**Table 9:** Energy assessment of chromium recovery from leather tanning residues using various impact categories associated with the CED approach (FU = 1 kg of chromium recovered).

Impact category	Unit	Total	Sodium hydroxide	Tap water	Energy production from biogas
Total	MJ	2.61E + 01	2.56E + 01	3.14E-01	1.83E-01
Non-renewable, fossil	MJ	2.00E + 01	1.97E + 01	1.82E-01	1.46E-01
Non-renewable, nuclear	MJ	3.50E + 00	3.39E + 00	9.41E-02	2.03E-02
Non-renewable, biomass	MJ	1.89E-03	1.86E-03	1.55E-05	1.21E-05
Renewable, biomass	MJ	6.73E-01	6.60E-01	6.70E-03	7.21E-03
Renewable, wind, solar, geothermal	MJ	4.96E-01	4.81E-01	1.20E-02	3.12E-03
Renewable, water	MJ	1.48E + 00	1.46E + 00	1.85E-02	6.77E-03

Table 9 also highlights the total energy demand and the contribution of different energy sources, both renewable and non-renewable, towards chromium recovery. Non-renewable energy sources, particularly fossil fuels, dominate the energy demand, constituting a significant portion of the total energy consumption. Fossil fuels, non-renewable biomass and nuclear energy, collectively contribute to approximately 90% of the total energy demand. This heavy reliance on non-renewable sources underscores the current challenges in achieving sustainability in chromium recovery processes. In contrast, renewable energy sources, such as biomass, wind, solar, geothermal, and water-based energy, contribute a smaller but notable percentage to the overall energy demand. While their contribution is comparatively lower, the inclusion of renewable energy sources, especially in the production of sodium hydroxide, signifies a step towards mitigating environmental impacts and promoting sustainable practices in chromium recovery.

A standout feature of this study is the innovative approach to energy recovery for heating and electricity from leather residues, turning waste into a valuable resource. By producing biogas from these residues, the study showcases an eco-friendly alternative to traditional natural gas, significantly cutting down on pollution. Without this energy recovery, the results would have been less favorable. Utilizing biogas as an energy source further reduces fossil-based energy consumption as well as unfavorable emissions, demonstrating a sustainable and impactful solution that benefits the environment and enhances the overall sustainability of the leather industry. In addition to chromium recovery, these energy recovery methods underscore the potential for comprehensive waste valorization, contributing to a circular economy and further mitigating the environmental impact of leather production.

**Fig. 9:**
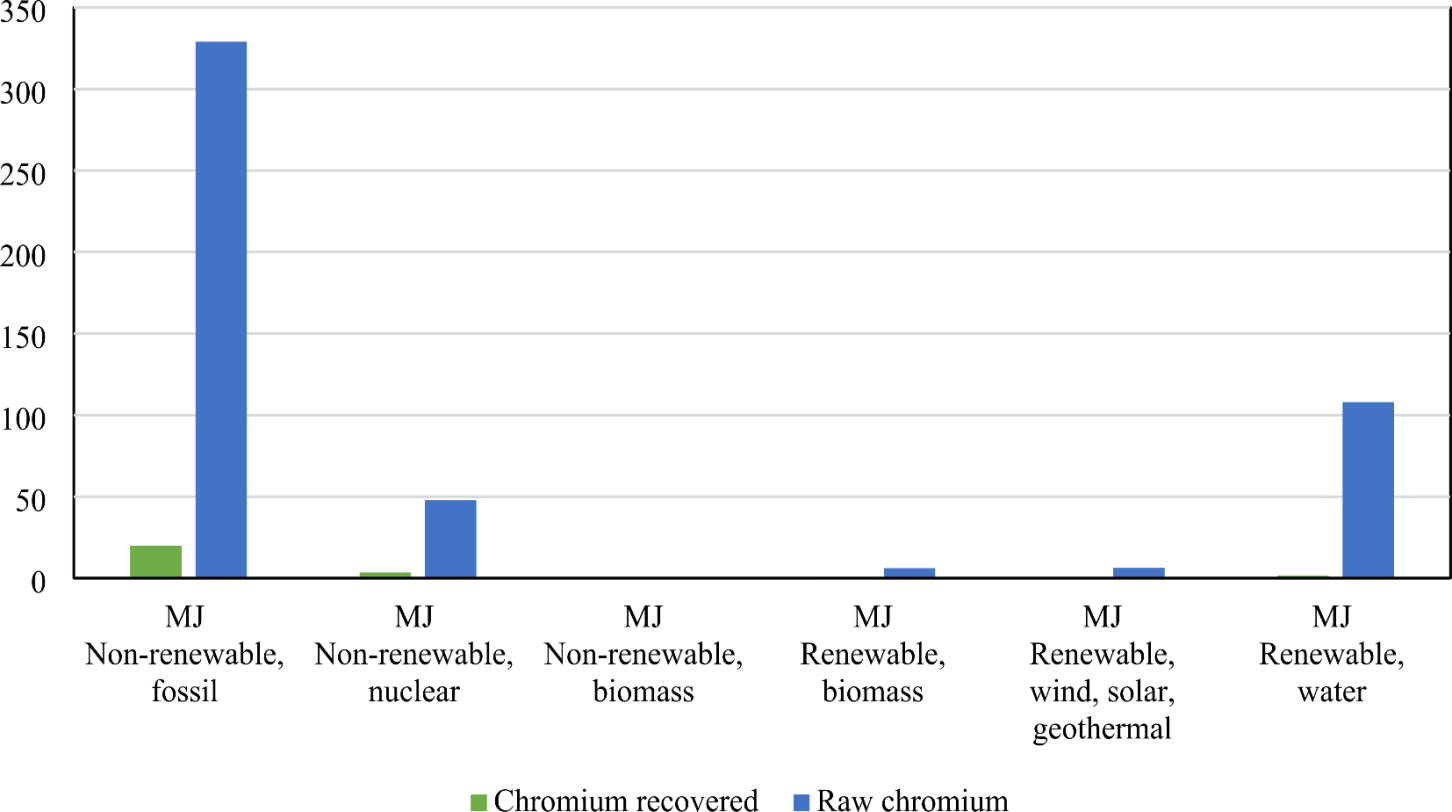
Comparative CED of recovered chromium vs. raw chromium production.

Despite chromium recovery relying predominantly on renewable resources, the cumulative environmental burden over its lifetime remains significantly smaller compared to crude chromium production (Fig. 9). This finding underscores the potential environmental benefits and sustainability advantages of adopting chromium recovery processes. Overall, the comparison presented in Fig. 9 underscores the importance of embracing sustainable alternatives, such as chromium recovery from leather tanning residues, to mitigate environmental impacts and promote long-term ecological resilience in industrial processes.

**Fig. 10:**
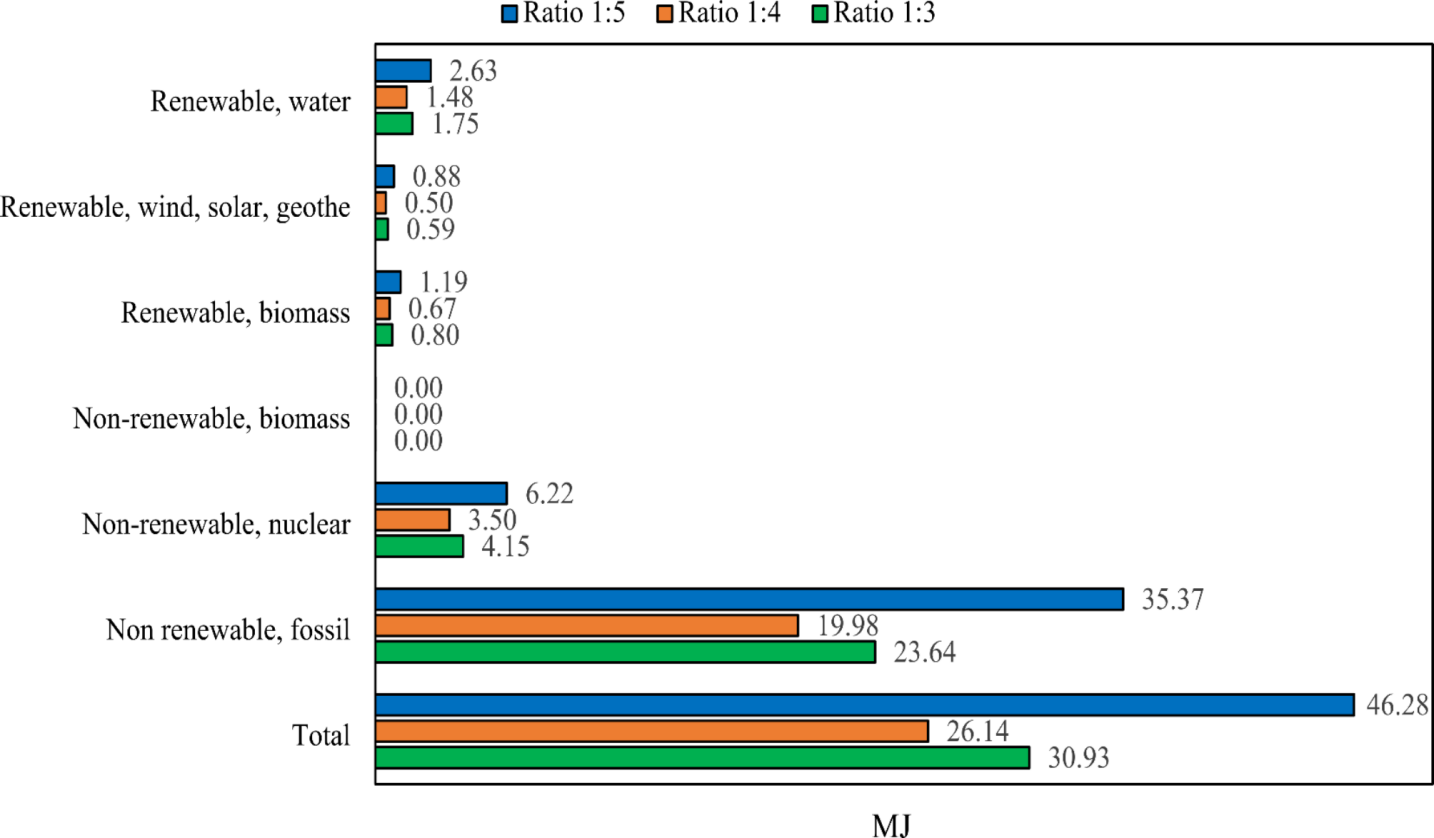
Sensitivity analysis of environmental impacts for chromium recovery: comparative assessment of 1:3, 1:4, and 1:5 ratios across CED.

Figure 10 also illustrates the results of the sensitivity analysis for CED based on the concentration of sodium hydroxide. The findings indicate that increasing and decreasing the concentration of sodium hydroxide leads to a greater removal of inherent energy from nature during the chromium reactor process.

## Conclusions

While leather industries hold immense appeal for their utilization of livestock byproducts to create value-added materials, they grapple with significant pollution challenges, particularly from heavy metals like chromium present in their waste streams. Addressing this issue is pivotal for enhancing the environmental sustainability of the industry. Fortunately, innovative methods have emerged to recover chromium from these waste streams, offering not only economic benefits but also crucially preventing its contamination of water and soil sources. Moreover, these methods can be extended to promote a circular economy by harnessing the potential of biogas production, energy generation, and nitrogen fertilizer synthesis from the residual materials. To ensure the viability and sustainability of these approaches, it is imperative to conduct comprehensive environmental and energy assessments using methodologies like LCA. This study aims to delve into precisely this aspect, focusing on the environmental and energy recovery aspects of chromium from leather tanning waste. Beyond merely energy and fertilizer production, the research seeks to explore the broader implications of these interventions for enhancing the circularity and sustainability of the leather industry. Here are several key discoveries from the research:

- Based on the results of physical and chemical tests, it was found that the physical and chemical characteristics of leathers tanned with the addition of chromium recovered from waste are similar to those of traditionally tanned leather.
- Resource damage amounts to $8.42E-02 per kg of chromium recovered, while human health and ecosystem damages are measured at 4.28E-06 DALY and 1.60E-08 species.yr per kg of chromium recovered, respectively.
- Through a weighted environmental impact assessment, the study quantifies the overall environmental burden of chromium recovery, with a total weighted environmental impact of 201.04 mPt per kg of chromium recovered.
- A comparison reveals notable reductions in environmental damage when comparing recovered chromium from leather tanning residues to raw chromium production. For example, recovered chromium shows reductions of 95.65% in total environmental damage, 93.05% less damage to human health, 93.25% less damage on ecosystems, and 97.47% less damage on resources.
- The total energy demand for recovering 1 kg of chromium is 2.61E + 01 MJ. Non-renewable sources, mainly fossil fuels, constitute 90% of energy use, underscoring sustainability challenges.
- Sodium hydroxide emerges as a major contributor to environmental impacts and damages, particularly in resource depletion and human health impacts. It also accounts for 98% of the total energy demand during chromium recovery. Future research should focus on investigating the use of less environmentally damaging chemicals as substitutes for sodium hydroxide in the chromium recovery process, exploring the integration of renewable energy sources to reduce reliance on fossil fuels, conducting long-term impact studies to evaluate the sustainability of chromium recovery technologies, and performing detailed economic analyses to assess the cost-effectiveness of these technologies compared to traditional waste management practices.
- The sensitivity analysis revealed that both increasing and decreasing the sodium hydroxide concentration significantly affect both environmental impact and CED. Higher concentrations, particularly at the 1:5 ratio, lead to a substantial increase in environmental impacts (nearly 80%) and higher energy demand, indicating that while the process may improve separation efficiency, it also increases the environmental burden. Moreover, the 1:3 ratio showed a moderate increase of around 20% in environmental impacts.

Finally, policy recommendations include providing subsidies or tax incentives to encourage chromium recovery technologies, establishing industry standards for chromium recovery and recycling, increasing funding for research into alternative chemicals and renewable energy applications, and raising awareness about the environmental and economic benefits of chromium recovery. Addressing these areas can help the leather industry reduce its environmental footprint and enhance sustainability.

## Data Availability

The datasets generated and/or analyzed during the current study are available in the Leather_waste_Hydrolysis repositoryhttps://github.com/shimashimashafeie73/LCA.
